# Polyarticular Neurogenic Heterotopic Ossification in a Spinal Cord Injury: A Case Report from Saudi Arabia

**DOI:** 10.7759/cureus.4019

**Published:** 2019-02-05

**Authors:** Taha Ali, Rouaa F Mandurah, Sami Ullah

**Affiliations:** 1 Physical Medicine and Rehabilitation, King Fahad Medical City, Riyadh, USA; 2 Physical Medicine and Rehabilitation, King Fahad Medical City, Riyadh, SAU

**Keywords:** heterotopic ossification, spinal cord injury, traumatic brain injury, activities of daily living, bilateral hip joints, bilateral knee joints

## Abstract

A 33-year-old male victim of a motor vehicle accident, who presented with a T12 (thoracic 12 vertebra) burst fracture (ISNCSCI T11 AIS-A: International Standards for Neurological Classification of Spinal Cord Injury T11 ASIA Impairment Scale), was admitted to a rehabilitation hospital. A stage-II left ischial pressure ulcer was also reported. An X-ray of the pelvis revealed bilateral neurogenic heterotopic ossification (NHO) in both hips and knees, which was further confirmed by TC-99m methylene diphosphonate (MDP) bone scintigraphy. Interventions included indomethacin and conservative management. Surgery was not preferred, as NHO was still immature. Moreover, patient transfer and lower body dressing were unaffected by NHO. It is important to consider an early radiological screen in selected high-risk cases for NHO, to minimize the risk of associated complications.

## Introduction

Spinal cord injury (SCI) is a neurological disorder that not only disrupts motor and sensory signaling across the injury site but also affects the functioning of the autonomic nervous system [1]. SCI often leads to disabilities that hinder the activities of daily living [2]. Huge efforts have been made by researchers in understanding the pathogenesis and early recognition and treatment of SCI; still, it remains a devastating disorder [3]. The incidence of traumatic SCI (TSCI) in Middle East countries is under-estimated, and the probable estimates are 15 TSCI per million per year [4]. The rate of traumatic SCI caused by land transport accidents in Saudi Arabia is reported to be 85%, the highest in the world [5]. However, there is no data registry system available to collate the whole TSCI data in Saudi Arabia. Introducing a proper data registry system may help in closely knowing the status of TSCI etiology [6]. A recent study reported the prevalence of NHO in patients with TSCI to be 11% [7].

Additionally, it can be found in patients with hip surgery, burns, stroke, encephalopathy, and cerebral palsy or it can be hereditary, like osteodystrophy [8]. The most commonly affected joint is the hip but other locations include the knee, elbow, and shoulder [9]. People with SCI develop significant restriction in the range of motion (ROM) that interferes with mobility and the activities of daily living (ADLs) [2]. Bilateral hip and knee NHO following SCI is rare and has been rarely reported before in the literature.

## Case presentation

**Figure 1:**
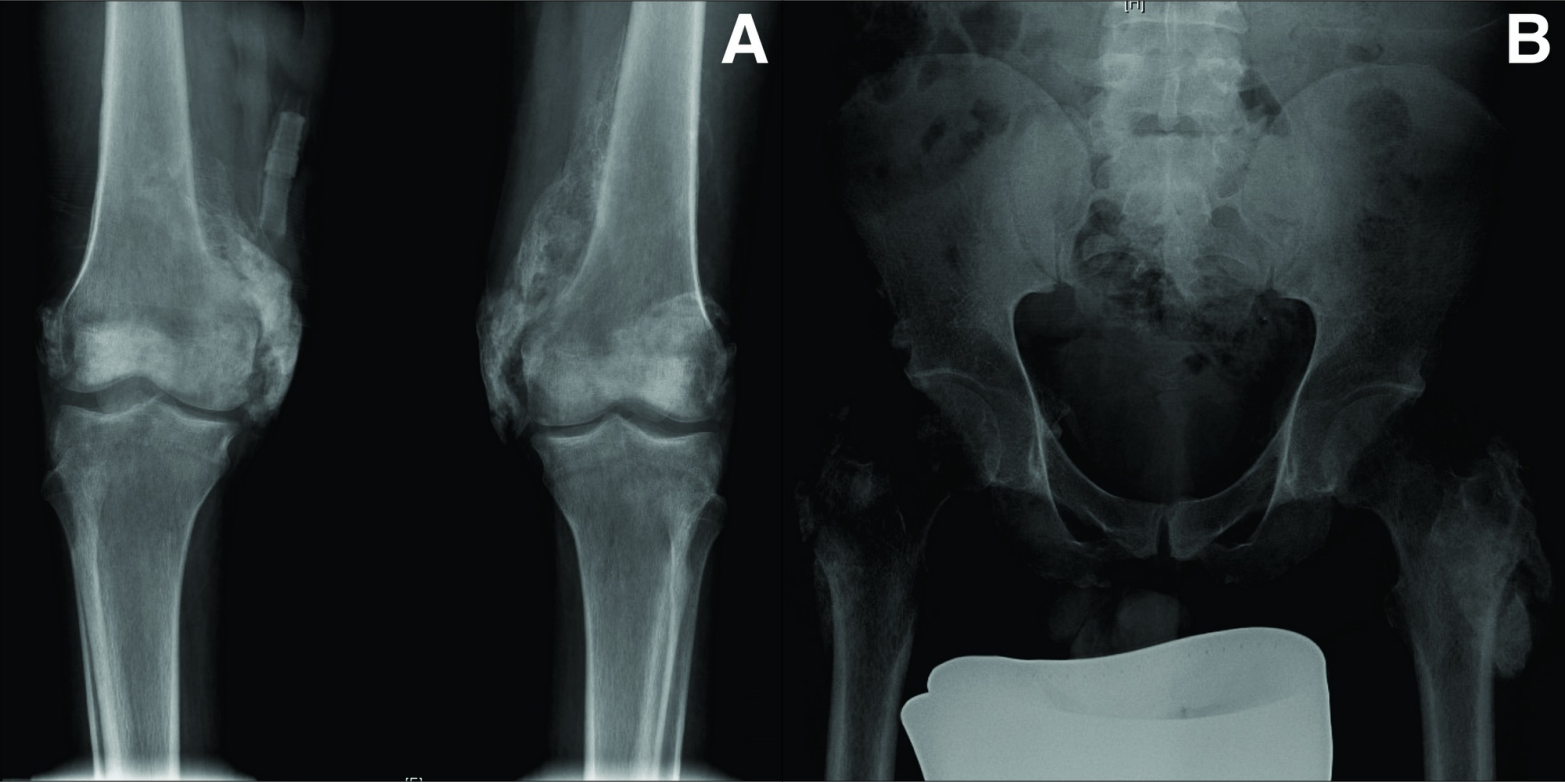
Mineralized bone visible around both knee and hip joints

**Figure 2:**
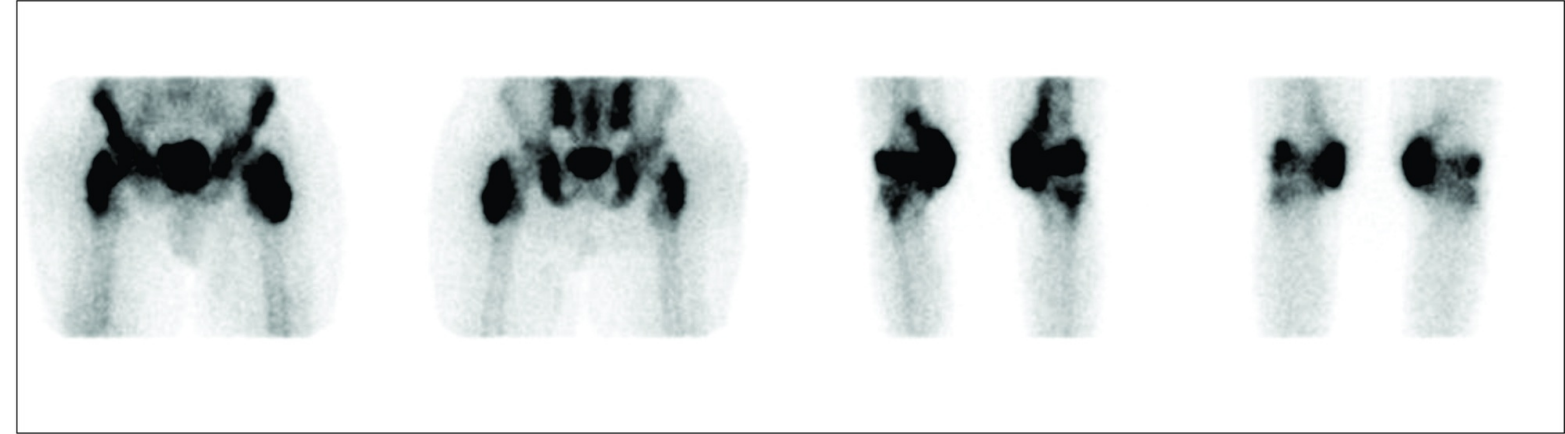
TC-99m MDP bone scintigraphy: increased intake around both hips and knees MDP: methylene diphosphonate

A 33-year-old male involved in a road traffic accident (MVA) in July 2016 was admitted to our hospital. He sustained a T12 burst fracture, thus requiring open reduction and internal fixation from T10-L1. As a result of SCI, he developed paraplegia (T11 AIS-A), a pressure injury, and double incontinence. Upon admission to the rehabilitation hospital, he was found to have a left ischial pressure ulcer (stage II). The pelvis X-ray showed bilaterally symmetrical NHO in both hips (Figure 1A) and knees (Figure 1B). Subsequently, TC-99m methylene diphosphonate (MDP) bone scintigraphy (Figure 2) showed findings compatible with NHO around both knees and hip joints bilaterally, and it appeared to be immature. He was managed conservatively and commenced on indomethacin. The surgical referral was not considered, as the NHO was not affecting the patient’s transfers, lower body dressing, seating, skin, and other aspects of daily living.

## Discussion

NHO is a frequent complication following SCI. It is defined as the abnormal formation of lamellar bone inside soft-tissue structures [10]. The exact pathophysiology of NHO is still not clear. However, multiple theories indicate disturbed neuronal activity along with prolonged immobilization, tissue hypoxia, and hypercalcemia. Thus, various humoral, neural, and local factors are held responsible behind the heterotopic ossification (HO) pathophysiology [11]. The usual clinical symptoms associated with HO include local joint pain, muscle pain, limited ROM, reduced motility of hip joint, and swelling. These may occur after three to 12 weeks of injury [12]. NHO is reported to grow more and completely evolve within two years after the injury [7].

In our case, clinical examination suspected NHO on admission to the spinal rehabilitation unit, and this was subsequently confirmed radiologically. The NHO was still immature at the time of admission to the rehabilitation unit. Thus, it is important to screen such cases earlier, to minimize the risk of complications associated with NHO. NHO usually occurs below the level of a central nervous system (CNS) lesion, and the complete transverse SCI goes parallel with the severity of NHO [11,13]. We started oral indomethacin, a non-steroidal anti-inflammatory drug (NSAID), in our study patient. It has already been reported in previous studies that inflammation is a key factor involved in NHO development, and, therefore, some have hypothesized that the incidence and impact of NHO may be reduced with NSAIDs treatment. Some of the preliminary studies have reported the reduced occurrence of NHO in SCI patients treated with indomethacin [14-15]. For established NHO, surgical excision is considered as the only effective intervention available presently [16]. A recent systematic review of the surgical resection of SCI-induced NHO recommends earlier intervention once comorbid factors are controlled [16-17]. The surgical process is not preferred when nerves and blood vessels are entangled by ectopic bone. Around 20% of the cases of NHO are reported to relapse, with several other conditions like perioperative fracture and infections [18-19]. Therefore, we did not pursue the surgical option in this case. ADLs in patients with NHO can be affected secondary to pain, reduced ROM of the involved joint, poor posture, and the associated increased risk of a pressure injury. Prophylaxis includes the management of risk factors, such as spasticity, urinary tract infections (UTIs), decubitus ulcers, and deep vein thrombosis (DVT), which may reduce the risk of developing NHO [20]. It is recommended to cautiously follow the practice of consistently moving the large peripheral joints (gentle range of motion) after the injury. This helps in preventing muscle shortening and maintaining joint flexibility [11]. NHO in the bilateral hip joints and bilateral knee joints associated with SCI has never been reported previously and its pathogenesis is also unknown. This has further led to the lack of consensus on treatment modalities. As NHO affects ADL in patients with SCI, early diagnosis, prevention, and consensus on treatment modalities desperately need to be explored.

## Conclusions

NHO in the bilateral hip joints and bilateral knee joints associated with SCI has never been reported previously and its pathogenesis is also unknown. This has further led to the lack of consensus on treatment modalities. As NHO affects ADL in patients with SCI, early diagnosis, prevention, and consensus on treatment modalities desperately need to be explored.
